# Clinical, Socioeconomic, and Psychosocial Factors Associated with Blood Pressure Control and Adherence: Results from a Multidisciplinary Cardiovascular National Program Providing Universal Coverage in a Developing Country

**DOI:** 10.1155/2018/5634352

**Published:** 2018-07-09

**Authors:** Daniela Sandoval, Carolina Nazzal, Tomás Romero

**Affiliations:** ^1^Department of Primary Care & Health Family, Faculty of Medicine, University of Chile, Santiago, Chile; ^2^Department of Physical Therapy, Faculty of Medicine, University of Chile, Santiago, Chile; ^3^School of Public Heath, Faculty of Medicine, University of Chile, Santiago, Chile; ^4^School of Medicine, University of California, San Diego, CA, USA; ^5^Institutional Review Board, Sharp Health Care, San Diego, CA, USA

## Abstract

**Background:**

Limited information exists on blood pressure (BP) control factors and adherence to antihypertensive drug therapy (Rx) in developing countries.

**Methods:**

Cross-sectional study in randomly selected 992 hypertensive patients under a Chilean national comprehensive Cardiovascular Health Program (CVHP). Association of education, income, diabetes, obesity, physical activity, psychosocial characteristics, smoking, and alcohol abuse with BP control and adherence were evaluated by multivariate logistic regression.

**Results:**

BP control (<140/90 mmHg) was achieved in 63.1% of patients, with 38.4% adherent to Rx. Uncontrolled BP significantly associated with male sex (OR: 1.73 [95% CI 1.35-2.22]), low family income, high emotional-stress-depression score, body mass index, no adherence (OR: 1.83 [95% CI 1.44 - 2.32]), multiple Rx, baseline systolic BP value, and sedentary life style. Males (OR: 1.54 [95% CI 1.23 - 1.93]), low family income, high emotional stress-depression score (OR: 2.15 [95% CI 1.68 - 2.76]), low social support, and uncontrolled BP (OR: 1.52 [95% CI 1.22-1.90]) associated with no adherence.

**Conclusions:**

Comparable BP control (63.1%) to higher-income societies was observed. Uncontrolled BP associated significantly to no adherence and both to male sex, socioeconomic, and psychosocial factors. Global low adherence (38.4%) and improved BP control and adherence in diabetics were noted.

## 1. Introduction

Unsatisfactory BP control according to accepted guidelines is a persistent worldwide problem in the hypertensive population [1, 2]. In high-income countries approximately 55% of patients with access to antihypertensive drug therapy (Rx) do not reach a satisfactory BP control (<140/90 mmHg), and lack of adherence to treatment (Rx) is considered an important factor [3–5]. A World Health Organization sponsored study (SAGE) that included 47,443 adults from six middle-income countries (China, Ghana, India, Russia, Mexico, and South Africa) sampled between 2007 and 2010 documented that more than 90% of the hypertensive patients had uncontrolled BP, and insurance as well as income status emerged as significant correlates to diagnosis and treatment [6]. Unfavorable socioeconomic and psychosocial conditions have been frequently mentioned as barriers for BP control and adherence to Rx, of particular relevance in developing countries and/or social groups with limited access to health resources [6–10].

Chile is a developing country undergoing an accelerated process of socioeconomic transition and classified along with Uruguay in the high-income bracket in South America yet with one of the highest coefficients of social inequality (GINI Index 50.5) [11].

Approximately 75% of the Chilean population receives primary medical care in government sponsored primary care centers financed through FONASA (Fondo Nacional de Salud, National Health Fund) a legally mandated 7% tax on wages and from government subsidies (64% of total)[12]. Approximately 20% of the populations are affiliated to private or institutional medical services, with a different financing (private) system. FONASA guarantees coverage free of copayments for medical care at government sponsored primary care centers including approved medications for eighty medical conditions through a Health Care Policy Act approved in 2005 (Garantías Explícitas de Salud, GES, or Explicit Health Warranties) [13].

In 2002 a Cardiovascular Health Program (CVHP) (*Programa de Salud Cardiovascular*) was started in Chile implemented at the government sponsored primary care centers as part of the National Health Program aimed at reducing cardiovascular morbidity, the mayor cause of death and the third cause of disability in Chile today [10]. The increasing rates of obesity and diabetes in the Chilean hypertensive population were major determinants of this program [10, 14]. In the CVHP patients are provided access to treatment and screening for cardiovascular risk factors plus a multidisciplinary approach to modify unfavorable life style and habits free of copayments. It is estimated that approximately 500,000 hypertensive patients are followed through the CVHP in the Metropolitan region of Santiago [14]. Previous results from hypertensive patients evaluated between 2009 and 2010 enrolled in the CVHP since 2006 have been published [10]. The objective of the present study is to evaluate the association of socioeconomic and psychosocial factors, life style behavior, and adherence to Rx with BP control, not analyzed in previous studies, in 992 patients from four different primary care centers with at least one year attending the CVHP.

## 2. Methods

### 2.1. Study Population

The study was conducted in four government subsidized primary care centers in the Metropolitan region of Santiago, Chile. Patients at the primary care centers with BP ≥140/90 mmHg are referred to the CVHP and screened for associated cardiovascular risk factors (diabetes, dyslipidemias, and renal failure). Approximately every 3 months they were followed by a team of primary care physicians, dietitians, and trained medical assistants providing clinical evaluation, assessment of life style habits, and counseling for modification of unhealthy behavior.

Hypertensive patients followed in the selected centers were considered for inclusion in the study; a randomly selected sample of 992 was obtained from 14,363 patients under care in these centers. Data was collected between 2011 and 2014. The sampling size was estimated for an expected prevalence of BP < 140/90 mmHg of 50%, with 95% of confidence, estimated error of 5%, and design effect of 2.5. None of these patients have been part of a previous study completed between 2009 and 2010 in different primary care centers in the Santiago Metropolitan Region.

Inclusion criteria considered patients between 30 and 64 years of age, who had completed at least a year in the CVHP receiving antihypertensive drug therapy at the time of recruitment. Patients with significant disabilities (bedridden, mentally incompetent, and wheel chair users), pregnant women, or with frequent unjustified missed appointments were excluded (n=17). Excluded patients were replaced according to a random procedure using the EPI-Info software.

**Figure 1:**
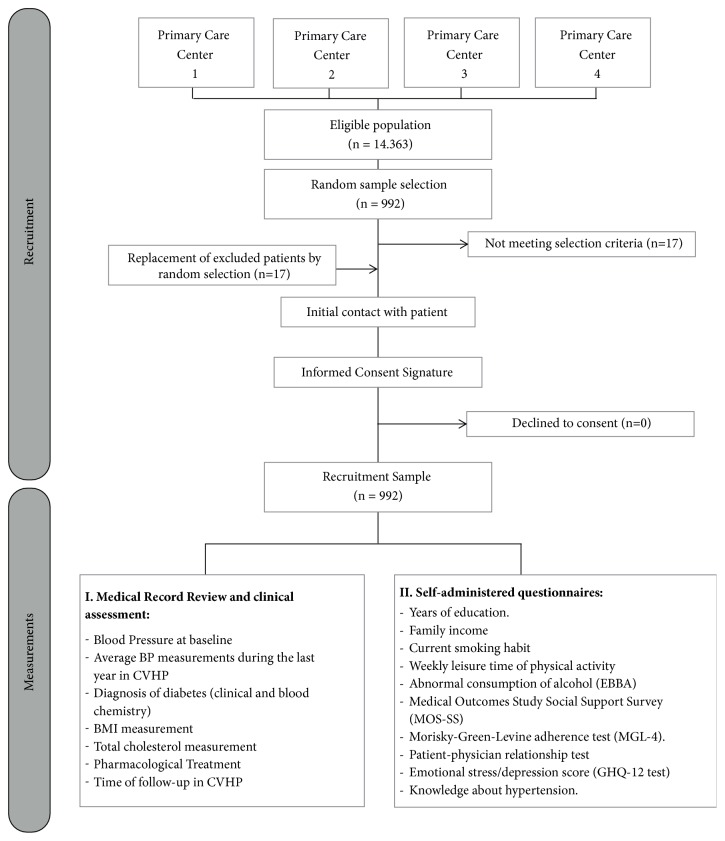
Recruitment process and procedures followed after patient's agreement to participate in the study.

Patients were invited to participate in the study by trained medical assistants working at the primary care centers. It was explained to patients that their participation only implied an agreement to authorize the information collected to be analyzed and eventually published without compromising their privacy and ensuring confidentiality. If initial hesitation from patients to participate was encountered, follow-up of contacts was carried out to address all their concerns. Finally, all patients initially invited consented to participate (Figure 1).

Patients with documented associated cardiovascular comorbidities (coronary heart disease, valvular heart disease, and rhythm disturbances) or requiring hemodialysis were referred to secondary (specialist) level of care before randomization and therefore not included in the study (3.17% of the eligible population, 14,363 patients).

### 2.2. Variables

513 patients were recruited between August 2011 and June 2012 and 479 between August 2013 and June 2014. At the time of recruitment demographic, education, income, clinical, weight, and height data was obtained and questionnaires previously validated in compatible Chilean populations regarding adherence, psychosocial variables, and life style habits (smoking, use of alcohol, and physical activity) were filled at the clinic or within a week by the patients who preferred to do it at home; assistance to clarify questions was always available as requested. The blood chemistry values used in the analysis of the study were the most recent ones available at the time of recruitment. Data analysis was completed on 07/2016.

Trained medical assistants recorded BP values at each one of the patient's visits after three successive measurements with the patient sitting at least for 5 minutes using mercury sphygmomanometers and recording the average of the two last measurements. The BP value at the time of referral to the CVHP was interpreted as the baseline BP and the average of BP measurements recorded during the last year of follow-up preceding the time of recruitment in the study was considered the final BP results. The average time of care in the CVHP was 7.5 ± 4.6 years. Presence of diabetes and other comorbidities was established by clinical diagnosis and/or therapies prescribed. Blood chemistries that included fasting blood glucose, total and HDL cholesterol, and HbA1 in diabetics were measured using standard techniques. Weight was measured using a calibrated digital scale with patients in underwear and barefoot. Body mass index (BMI) was calculated dividing the weight in kilograms by the square of height in meters. All the antihypertensive medications in use at the time of recruitment were recorded.

Family income was defined as the ratio between the total monthly family income and total number of members in the family. Low family income was defined as < than the lower quartile of monthly income for individuals (< US$ 80/per person). Low education corresponded to < than 8 years of schooling.

### 2.3. Demographic and Psychosocial Characteristics Measurements

1. The Morisky-Green-Levine (MGL-4) [15, 16]: a four-item questionnaire was utilized to assess adherence to Rx. A positive answer = 1 point; a negative response = 0 point. A patient is considered no adherent with a score of 1 or higher. This test has an alpha Cronbach value of 0.61 for controlled BP predictive value at 5 years [16]. A sensitivity analysis considering a score of 2 or higher for no adherence was carried out and results are included in Supplemental Table (available here) and discussed in results.
2. MOS-SS (Medical Outcome Study Social Support Survey) [17], adapted to Spanish and validated for its use in Chilean primary care. It collects multidimensional information about the levels of support that the patient can access.
3. Knowledge about hypertension: the following questionnaire was utilized:* Is hypertension a life-long condition? Is it possible to control it by diet and medications? Mention two or more organs that may be damaged by high BP*. An incorrect answer to any of these questions is indicative of low knowledge.
4. Emotional Stress/ Depression score: A Chilean validated Spanish version of the GHQ-12 questionnaire was utilized [18]. This test detects and grades the levels of emotional stress and depression.
5. Patient-physician relations: a version of a Chilean validated questionnaire by Bozzo-Martinez [19] to assess the patient satisfaction with care received at the primary care center was utilized. A score of 71 or less was considered indicative of inadequate patient-physician relation.
6. Smoking: smokers were defined as those currently smoking at the time of the study.
7. Use of alcohol: it was evaluated using the EBBA [20] survey (Brief Survey of Abnormal Drinking) to identify alcohol related abnormal behavior, previously validated and of common use in Chile [21].
8. Physical activity: physically active patients were defined as those who electively exercised for at least 30 minutes three times a week [21].

Using currently accepted clinical guidelines we classified controlled BP as < 140/90 mmHg, elevated total cholesterol as ≥ 200 mg/dl, and obesity as body mass index ≥ 30 kg/m^2^. Multiple antihypertensive drug therapy was defined when patients were prescribed 2 or more drugs.

The Ethic Committee of Research in Humans of the University of Chile Faculty of Medicine approved the study protocol.

### 2.4. Statistical Analysis

Exploratory analysis for the continuous variables was performed. Categorical variables were expressed as absolute or relative frequencies. Continuous variables were measured as medians (percentile 25 and 75). Differences according to sex, BP control, and adherence for categorical variables were analyzed using the* X*^***2***^ test and Mann–Whitney test for continuous variables; p value <0.05 was considered a significant difference.

For uncontrolled BP (≥ 140/90 mmHg) odds ratio (OR) with 95% of confidence intervals was calculated. In Model 1 (unadjusted model) a univariate logistic analysis was performed in reference to uncontrolled BP, which included age (years), baseline systolic and diastolic BP (mmHg), time of care in the CVHP (years), and BMI (kg/m^2^) as continuous variables and sex (male), low education (< 8 years of educations), low family income (< 80 US$ per person/month), inadequate patient-physician relation (score ≤ 71), high emotional stress-depression (score ≥7), low social support (score <57), low knowledge about hypertension (score ≥1), no adherence (score ≥ 1), multiple antihypertensive RX (≥ 2 drug antihypertensive), diabetes mellitus, elevated total cholesterol (≥ 200 mg/dL), smoking (current), alcohol related abnormal use (score ≥2), and sedentary behavior (<30 minx3/week of leisure physical activity) as dichotomous variables.

In Model 2, all variables that were statistically significant at the p<0.05 level in the unadjusted model were included in the multivariable logistic regression analysis. For no adherence a similar univariate logistic analysis was conducted (Model 1), using in the multivariable logistic regression analysis (Model 2) all the univariate variables that showed statistical significance at the p<0.05 level.

## 3. Results

### 3.1. Demographic, Socioeconomic, Clinical, and Antihypertensive Therapy and Anthropometric Characteristics

A larger number of women (n=647, 65.2%) than men (n=345, 34.8%) were part of this study. Low family income and low education level were documented in 23.0 and 33.2%, respectively. Obesity (BMI ≥ 30 kg/m^2^) was found in 58.4% of patients; higher in women than men (61.1% versus 53.3%, p=0.019), and 37.8% were diabetics (42.3% men, 35.4% women, p=0.032) (Table 1).

The baseline median BP value was 146/91 mmHg. The median BP value corresponding to the last year of care in CVHP at the time of recruitment was 132/80 mmHg. BP control was achieved in 63.1% of patients (66.0% women versus 57.7% men, p=0.01). Adherence to Rx was 38.4%, greater in women (41.1% versus 33.3%, p= 0.016). Alcohol abnormal behavior was more frequent in men (25.5% versus 7.4%, p<0.001); no sex differences were observed in current smoking (25.4%) and sedentary life style prevalence (83%). Multiple drug therapy was prescribed in 46.9% (41.2% men and 49.6% women, p= 0.011).

### 3.2. Factors Associated with Uncontrolled Blood Pressure

Male sex (OR: 1.73 [95% CI 1.35 - 2.22]), low family income, high emotional-stress-depression score (OR: 1.32 [95% CI 1.03 - 1.70]), BMI, no adherence (OR: 1.83 [95% CI 1.44 - 2.32]), multiple antihypertensive drug therapy (OR: 1.53 [95% CI 1.21 - 1.94]), baseline systolic BP value (OR: 1.03 [95% CI 1.02 - 1.04]), and sedentary life style (OR: 1.61 [95% CI 1.15 - 2.25]) were significantly associated with uncontrolled BP in Model 2 (adjusted model). The time of care patients received in the CVHP was not predictive of uncontrolled BP in Model 2 (Table 2).

### 3.3. Factors Associated with No Adherence to Prescribed Antihypertensive Medications

No adherence was significantly associated with uncontrolled BP (OR: 1.52 [95% CI 1.22 - 1.90]), male sex (OR: 1.54 [95% CI 1.23 - 1.93]), low family income (OR: 1.39 [95% CI 1.11 - 1.72]), high emotional stress-depression score (OR: 2.15 [95% CI 1.68 - 2.76]), and low social support in Model 2 (adjusted). The time of care under the CVHP showed no association with no adherence in the univariate logistic analysis (Model 1). In contrast, diabetes mellitus (OR: 0.81 [95% CI 0.66 - 0.99]) was significantly associated with better adherence (in Models 1 and 2; Table 3).

It is noteworthy the associations of no adherence with uncontrolled BP, male sex, and psychosocial factors did not change after a sensitivity analysis using a no adherence MGL-4 score 2 or greater (Supplementary Table).

## 4. Discussion

The BP control (63.1%) observed in this sample of hypertensive patients enrolled in the CVHP compares favorably with results published in many high-income countries [22–25]. In addition, our results contrast with a 2004 study in a Chilean Southern region urban population that showed BP control in 30.7% of 1,838 patients under antihypertensive therapy [26], and the 2010 Chilean National Health survey that reported BP control in 45.3% of hypertensive patients who informed to be under treatment [21]. More recently, a study from a CVHP cohort showed BP control of 59.7% [10]. The adherence to antihypertensive drug therapy in our study was only 38.4%, but there is no reliable information of adherence in Chilean hypertensive patients for comparison with results prior to the CVHP. The influences of adherence in the results of our study are illustrated by the significant association found of uncontrolled hypertension to no adherence, consistent with many previous observations [3, 5, 9, 22]. Although the use of the MGL-4 questionnaire may have possibly overestimated the actual no adherence, it is also conceivable that the comprehensive multidisciplinary support provided the CVHP may have partially offset the negative influence of low antihypertensive drug therapy adherence in our results. The lack of association in the time patients received care in the CVHP and uncontrolled BP suggests that the beneficial influence of the program in achieving BP control may begin early and do not fade with a longer permanence of patients in it. It is of note that our findings are similar to those of the ALLHAT trial in USA that documented BP control in 56.2% of the patients after 1 year of management [25] through a similar free universal comprehensive management as in the CVHP. In this study Hispanics had 20% greater odds of achieving BP control than Non-Hispanic Whites, reversing dramatically previously documented trends between these racial/ethnic groups in USA [24].

The significant association of male sex, low family income, high emotional-stress-depression, no adherence to treatment (Rx), high BMI, multiple antihypertensive drug therapy, baseline systolic BP value, and sedentary life style with uncontrolled BP in our study is in agreement with previous studies [3, 4, 6, 7, 9]. Also we found a significant association of uncontrolled BP with low social support and male sex, factors that were also significantly predictive of no adherence. Conflictive results in the association of sex with BP control in treated hypertensive patients have been published [27, 28]. However, a study done in 5 European countries, Canada, and USA that included 73,446 patients showed that BP control in hypertensive patients was 2-fold higher in women compared with men in Spain, Italy, Canada, and the United States [29]. It is of note that none of these studies analyzed the association of BP control and adherence, or with socioeconomic and psychosocial factors. To the best of our knowledge, no previous study has documented that no adherence to Rx in men may contribute to the reported differences in BP control with women. The inferior BP control in men may be likely related to their larger no adherence, which may be also a factor partially involved in their underrepresentation in our study. It is also conceivable that the comprehensive multidisciplinary services provided by the CVHP may contribute in reducing patients negative expectations about therapies (the nocebo [30] effects that induce a reduction of their efficacy and or tolerance) and eventually lead to an improvement in compliance. In addition, conflicts with their work activities are a well-known factor limiting access of men to health care, issue that is a challenge for the Chilean health care services. Nevertheless, the proportion of men in our study (women/ men = 1.87) showed a slight increase when compared to the ratio found in a previous study (2.15) from a similar population followed in the CVHP [10], possibly reflecting a favorable trend in the recruitment of men to the program (please see Table 4).

The association of low family income, high emotional stress/depression, and low social support in our study underscored the significance of socioeconomic and psychosocial factors in BP control and adherence [6, 8, 31–34]. We also found that low income was associated with no adherence, despite patient's unrestricted free access to health care and drug therapy but consistent with previous observations and indicative of the complex causal pathways and well-documented relations of socioeconomic position and health [6, 8, 31]. Our finding that adherence to (Rx) appeared not to be hampered but favored by the association with diabetes has been noted by others in a study in hypertensive patients receiving multiple drug therapy due to comorbidities [35]. As discussed by these authors, hypertensive patients with associated comorbidities may be more aware of being at a higher risk and then more willing to follow the recommended drug therapy. It is of note that we found no differences in BP control between diabetics and nondiabetics, in contrast to a previous study from a CVHP population that showed worse BP control in diabetics [10] (Table 4). Since adherence was only measured in our current and more recent study it is uncertain if this difference also reflected adherence improvements in diabetics that plausibly may have occurred in the interim. These changes are significant achievements in a population with such alarming rates of obesity and diabetes (58.4 and 37.5 %, respectively).

Our study has several limitations. The underrepresentation of men as compared to women (women/men=1.87) in this cohort is a persistent problem in the CVHP, although an improvement trend may be already occurring (Table 4). As discussed, it has been attributed to conflicts with labor activities of men in Chile, but also probably reflective of the greater no adherence to therapy found in men. It is uncertain if the results of this study are applicable to patients that stayed in the program for less than 1 year or were older than 64 years of age (not included in this study). Although hypertensive patients with cardiovascular comorbidities or undergoing hemodialysis were referred to a second level of care (specialist) and not followed in the CVHP, it is unlikely that their small proportion could have influenced our results (3.17% of the eligible population). Finally, physical activity was assessed throughout a questionnaire used in the 2010 Chilean National Health Survey [21] that only counted time spend in leisure type of exercise and did not consider physical activity involved in transportation or work (bicycle, walking), common in the study population. This may explain the high number of sedentary subjects found in our study (86.9%).

## 5. Conclusions

Our study emphasizes the importance of socioeconomic and psychosocial factors influencing BP control and adherence in hypertensive patients in a developing society. The underrepresentation of hypertensive men enrolled in the CVHP, their worse BP control, and adherence represents an important challenge to address in this program. Nevertheless, our findings underscore an important historical improvement in BP control in Chile, a developing country in a stage of advanced socioeconomic transition, that we attribute to the implementation of a comprehensive, multidisciplinary approach in the management of hypertensive patients (CVHP) providing free of copayments medical care, and counseling in lifestyle modifying changes, with results in BP control similar to some of those reported in high-income societies [25, 35, 36]. These favorable results were highlighted by the results noted in the hypertensive diabetic group, a particularly vulnerable population, with CVHP historical improvement in BP control and similar adherence as nondiabetic hypertensive. The lack of influence of time spend by patients in the program to achieve favorable BP results provides additional support to the consideration of programs similar to the CVHP in the developing world.

## Figures and Tables

**Table 1:** Demographic, socioeconomic, clinical, and antihypertensive therapy and anthropometric characteristics (n=992).

**Participant Characteristics**	**Total** (n= 992)	**Men** (n=345)	**Women** (n=647)	*p-value*
**Demographic and socioeconomic characteristics**				
Age (years)	56.0 (50 - 61) ^a^	57.0 (51 - 62) ^a^	55.0 (49 - 61) ^a^	<0.01
Low education (<8 years of education)	329 (33.2)	103 (29.9)	226 (34.9)	0.11
Low family income (<US$80 per person/month)	228 (23.0)	53 (15.4)	175 (27.0)	<0.001
**Psychosocial variables **				
Inadequate patient –physician relation^*∗*874^	202 (23.1)	63 (21.0)	139 (24.2)	0.28
High emotional stress-depression score	258 (26.0)	59 (17.1)	199 (30.8)	<0.001
Low social support	338 (34.1)	88 (25.5)	250 (38.6)	<0.001
Low family Cohesion	366 (36.9)	95 (27.5)	271 (41.9)	<0.001
Self-perception of health (poor-very poor)	177 (17.8)	48 (13.9)	129 (19.9)	0.018
Knowledge about hypertension	542 (54.6)	190 (55.1)	352 (54.4)	0.84
**Blood Pressure**				
Systolic blood pressure (mmHg)	132 (123.3 – 143.2) ^a^	133 (124.3 – 144.9) ^a^	131 (123.0 – 142.7) ^a^	0.033
Diastolic blood pressure (mmHg)	80 (75.0 – 86.7) ^a^	82.0 (76.7 – 80.0) ^a^	80.0 (75.0 – 86.5) ^a^	0.006
Blood pressure control (BP<140/90 mmHg)	626 (63.1)	199 (57.7)	427 (66.0)	0.010
Baseline Systolic blood pressure (mmHg)	146 (140 -160) ^a^	150 (140 – 160) ^a^	144 (140 – 160) ^a^	0.134
Baseline Diastolic blood pressure (mmHg)	91 (90 – 100) ^a^	92.0 (90 – 100) ^a^	90 (90.9 – 100) ^a^	0.270
Time of care in CVHP (years)	7.0 (4.0 – 10.7) ^a^	6.3 (3.5 – 8.9) ^a^	7.5 (4.3 – 11.4) ^a^	<0.001
**Clinical variables**				
Diabetes mellitus (clinical diagnosis)	375 (37.8)	146 (42.3)	229 (35.4)	0.032
Total Cholesterol (mg/dL)^*∗*865^	200 (172 – 230) ^a^	193.0 (166 – 227) ^a^	203 (177 – 232) ^a^	0.005
Elevated total cholesterol (≥ 200 mg/dL)^*∗*865^	439 (50.8)	138 (45.7)	201 (53.5)	0.031
**Antihypertensive treatment variables**				
Multiple antihypertensive drug therapy (≥ 2 drugs)	463 (46.9)	142 (41.2)	321 (49.6)	0.011
Adherence to pharmacological therapy	381 (38.4)	115 (33.3)	266 (41.1)	0.016
**Anthropometrics variables**				
Weight (Kg)	78.0 (68.4 – 89.7) ^a^	84.2 (76.0 – 96.1) ^a^	75.0 (66.0 – 84.9) ^a^	<0.001
Height (m)	1.57 (1.52 – 1.64) ^a^	1.67 (1.63 – 1.71) ^a^	1.53 (1.49 – 1.57) ^a^	<0.001
Body mass index (Kg/m^2^)	31.2 (28.0 – 34.9) ^a^	30.6 (27.8 – 33.9) ^a^	31.5 (28.2 – 35.7) ^a^	0.001
Obesity (BMI ≥ 30 Kg/m^2^)	579 (58.4)	184 (53.3)	395 (61.1)	0.019
**Lifestyle habits**				
Smoking (Current smoker)	253 (25.4)	87 (25.2)	165 (25.5)	0.88
Alcohol related abnormal behavior	136 (13.7)	88 (25.5)	48 (7.4)	<0.001
Sedentary (<30 minx3/week of leisure physical activity)	823 (83.0)	284 (82.3)	539 (83.3)	0.82

^a^ Median values (percentile 25–75) or n (%);  ^*∗*^number of patients in whom measurement was performed. All values included in Table 1 were determined at the time of recruitment to the study with the exception of baseline BP measurement indicative of values at the time of referral of patients to the CVHP. Systolic and diastolic BP and BP control at time of recruitment considers the average of the values in the last year at time of recruitment.

**Table 2:** Multivariable adjusted odds ratio for uncontrolled BP (≥140/90 mm/Hg) in hypertensive patients followed in the Cardiovascular Health Program.

	BP <140/90 mmHg n= 626 n (%)	BP ≥140/90 mmHg n= 366 n (%)	Model 1 Unadjusted Odds Ratio (CI 95%)	Model 2 Adjusted Odds Ratio*∗* (CI 95%)
**Demographic and socioeconomic characteristic**				
Age (years)	56 (49 - 61)^a^	58 (50 - 63) ^a^‡	**1.03 (1.01 – 1.04)**‡	1.01 (0.99 – 1.03)
Male sex	199 (31.8)	146 (39.9)‡	**1.56 (1.30 – 1.86)**‡	**1.73 (1.35 – 2.22)‡**
Low education (<8 years of education)	191 (30.5)	138 (37.7)†	**1.58 (1.31 – 1.90)‡**	1.21 (0.94 – 1.56)
Low family income (<US$80 per person/month)	189 (30.2)	124 (34.0)	1.19 (0.99 – 1.44)	**1.44 (1.13 – 1.84)‡**
**Psychosocial variables**				
Inadequate patient –physician relation	179 (31.1)	100 (33.4)	1.20 (0.99 – 1.47)	1.07 (0.84 – 1.37)
High emotional stress-depression score	153 (24.4)	105 (28.7)	**1.29 (1.06 – 1.57)**†	**1.32 (1.03 – 1.70)†**
Low social support	221 (33.7)	127 (34.7)	1.15 (0.96 – 1.38)	1.08 (0.85 – 1.37)
Low knowledge about hypertension	333 (53.2)	209 (57.1)	1.15 (0.97 – 1.38)	1.17 (0.93 – 1.47)
**Antihypertensive treatment variables**				
No adherence	360 (57.5)	251 (68.6)†	**1.67 (1.39 – 2.00)**‡	**1.83 (1.44 – 2.32)**‡
Multiple antihypertensive RX	255 (40.7)	280 (56.8)‡	**1.73 (1.45 – 2.07)**‡	**1.53 (1.21 – 1.94)**‡
Time of care in CVHP (years)	6.6 (4.0 – 9.9) ^a^	7.6 (3.9 – 11.5) ^a^†	**1.03 (1.01 – 1.05)**‡	1.00 (0.97 – 1.02)
Systolic blood pressure baseline in CVHP	140 (134.5 – 152) ^a^	150 (140 – 164.2) ^a^‡	**1.03 (1.03 – 1.04)**‡	**1.03 (1.02 – 1.04)‡**
Diastolic blood pressure baseline in CVHP	90 (90– 100) ^a^	96 (90 -100) ^a^‡	**1.02 (1.01 – 1.03)**‡	1.00 (0.99 – 1.01)
**Anthropometrics and clinical variables**				
Diabetes mellitus	248 (39.6)	127 (34.7)	0.88 (0.74 - 1.06)	0.87 (0.69 – 1.09)
Elevated total cholesterol (≥ 200 mg/dL)	289 (50.3)	150 (51.5)	1.01 (0.83 – 1.22)	1.07 (0.86 – 1.34)
BMI (Kg/m^2^)	30.8 (27.8 – 34.2) ^a^	32.1 (28.5 – 36.1) ^a^‡	**1.05 (1.03 – 1.06)**‡	**1.04 (1.02 – 1.07)**‡
**Lifestyle habits**				
Smoking (current smoker)	170 (27.2)	82 (22.4)	**0.74 (0.60 – 0.90)**‡	1.00 (0.78 – 1.30)
Alcohol related abnormal behavior	69 (11.0)	67 (18.3)‡	**1.69 (1.32 – 2.15)**‡	1.23 (0.89 – 1.71)
Sedentary	505 (80.7)	318 (86.9)‡	**1.60 (1.25 – 2.05)**‡	**1.61 (1.15 – 2.25**)‡

^a^ Median values (percentile 25–75); †p <0.05, ‡p <0.01. *∗*OR adjusted for all the variables that were significant at the p<0.05 level in unadjusted model: age (years), systolic and diastolic blood pressure baseline in CVHP (mmHg), and time of follow-up in CVHP (years) and BMI (Kg/m^2^) as continuous variables. Sex (male), low education (< 8 years of educations), high emotional stress-depression (score ≥7), no adherence (score≥ 1), multiple antihypertensive RX (≥ 2 drug antihypertensive), alcohol related abnormal (score ≥2), smoking (current), and sedentary (<30 minx3/week of leisure physical activity) as dichotomous variables.

**Table 3:** Multivariable adjusted odds ratio for no adherence risk to antihypertensive in hypertensive patients followed in the Cardiovascular Health Program.

	Adherence n= 381 n (%)	No Adherence n= 611 n (%)	Model 1 Unadjusted Odds Ratio (CI 95%)	Model 2 Adjusted Odds Ratio*∗* (CI 95%)
**Demographic and socioeconomic characteristic**				
Age	56 (50 - 61)^a^	55 (49 – 61)^a^	0.99 (0.98 – 1.00)	0.99 (0.98 – 1.00)
Male sex	115 (30.2)	230 (37.6)†	**1.47 (1.22 - 1.76)**‡	**1.54 (1.23 – 1.93)**‡
Low education (<8 years of education)	119 (31.2)	210 (34.4)	1.12 (0.93 - 1.35)	1.06 (0.85 – 1.32)
Low family income (<US$80 per person month)	68 (17.8)	160 (26.2)‡	**1.66 (1.35 – 2.06)**‡	**1.39 (1.11 – 1.72)**‡
**Psychosocial variables**				
Inadequate patient –physician relation	95 (28.1)	184 (34.3)†	**1.33 (1.09 – 1.63)**‡	1.04 (0.84 – 1.29)
High emotional stress-depression score	76 (19.9)	182 (29.8)‡	**1.84 (1.49 – 2.26)**‡	**2.15 (1.68 – 2.76)**‡
Low social support	113 (29.7)	225 (36.8)†	**1.44 (1.19 – 1.73)**‡	**1.34 (1.08 – 1.66)**‡
Low Knowledge about hypertension	200 (52.5)	342 (56.0)	1.08 (0.91 – 1.28)	0.98 (0.81 – 1.21)
**Antihypertensive treatment variables**				
Uncontrolled BP (≥ 140/90 mmHg)	160 (42.0)	305 (49.9)†	**1.67 (1.39 – 2.00)**‡	**1.52 (1.22 – 1.90)**‡
Multiple antihypertensive RX	197 (51.7)	266 (43.5)†	**0.81 (0.68 – 0.96)**†	0.92 (0.75 – 1.13)
Time of care in CVHP (years)	6.9 (3.9 – 11.9) ^a^	6.7 (3.9 – 10.2) ^a^	0.98 (0.96 – 1.00)	0.98 (0.95 – 1.00)
Systolic blood pressure baseline in CVHP	150 (140 - 160) ^a^	145 (140 - 160) ^a^	1.00 (0.99 – 1.00)	1.00 (0.99 – 1.00)
Diastolic blood pressure baseline in CVHP	94 (90 - 100)	90 (90 - 100)	0.99 (0.98 – 1.00)	1.00 (0.99 – 1.00)
**Anthropometrics and clinical variables**				
Diabetes mellitus	156 (40.9)	219 (35.8)	**0.80 (0.67 – 0.95)**†	**0.81 (0.66 – 0.99)**†
Elevated total cholesterol (≥ 200 mg/dL)	160 (48.3)	273 (52.7)	**1.23 (1.02 – 1.48)**†	**1.26 (1.04 – 1.54)**†
BMI (Kg/m^2^)	31 (28 - 35)^a^	31 (27 – 35) ^a^	1.00 (0.99 – 1.02)	1.00 (0.98 – 1.02)
**Lifestyle habits**				
Smoking (Current smoker)	97 (25.5)	156 (25.5)	0.98 (0.81 – 1.19)	0.98 (0.78 – 1.22)
Alcohol related abnormal behavior	39 (10.2)	97 (15.9)†	**1.55 (1.19 – 2.01)**‡	1.18 (0.86 – 1.60)
Sedentary	310 (81.4)	513 (84.0)	1.16 (0.92 – 1.45)	0.98 (0.75 – 1.29)

^a^Median values (percentile 25 – 75); †p <0.05, ‡p <0.01. *∗*OR adjusted for all the variables that were significant at the p<0.05 level in unadjusted model: sex (male), low family income <US$80 per person/month), inadequate patient-physician relation (score ≤ 71), high emotional stress-depression (score ≥7), low social support (score <57), uncontrolled BP (≥ 140/90 mmHg), multiple antihypertensive RX (≥ 2 drug antihypertensive), diabetes, elevate total cholesterol (≥ 200 mg/dL), and alcohol related abnormal (score ≥2) as dichotomous variables.

**Table 4:** Comparative values of BP control by sex, presence of diabetes, and adherence in the evaluation of two different groups from the CVHP.

	**2009 -2010 [10] ** **n = 1,194**	*p-value*	**2011-2014** **n = 992**	*p-value*
**Sex**				
Women	69.1 %	<0.01	65.2 %	<0.01
Men	31.9 %		34.8 %	
**BP according to sex**				
BP control in women	63.7 %	<0.01	66.0 %	0.01
BP control in men	52.4%		57.7 %	
**BP control and diabetes**				
BP control in diabetics	53.2 %*∗*	<0.01	66.1 %*∗*	0.12
BP control in nondiabetics	62.4 %		61.3 %	
**Adherence and diabetes **				
Adherence in diabetics	-** **-		41.6 %	0.10
Adherence in nondiabetics	-** **-		36.5 %	

*∗* p<0.01, difference between groups.

## Supplementary Materials

Supplementary Materials Supplementary Table: multivariable adjusted odds ratio for no adherence risk to antihypertensive drug therapy using Morisky-Green-Levine-4 items questionnaire in patients followed in the Cardiovascular Health Program. Association with outcomes using two different definitions of no adherence according to the score (> 1 versus > 2).
