# Supplementary material for: Clinical, Socioeconomic, and Psychosocial Factors Associated with Blood Pressure Control and Adherence: Results from a Multidisciplinary Cardiovascular National Program Providing Universal Coverage in a Developing Country
Source: Int J Hypertens. 2018 Jul 9;2018:5634352. doi: 10.1155/2018/5634352 (PMC6077571; doi:10.1155/2018/5634352)
Supplement: Supplementary Materials — Supplementary Table: multivariable adjusted odds ratio for no adherence risk to antihypertensive drug therapy using Morisky-Green-Levine-4 items questionnaire in patients followed in the Cardiovascular Health Program. Association with outcomes using two different definitions of no adherence according to the score (> 1 versus > 2). [file 5634352.f1.pdf]

Supplementary Table. Multivariable adjusted odds ratio for no adherence risk to antihypertensive drug therapy using Morisky-Green-Levine-4 items questionnaire in patients followed in the Cardiovascular Health Program. Association with outcomes using two different definitions of no adherence according to the score (> 1 vs. > 2).

	Odds Ratio* (CI 95%) for No adherence (Morisky score >1) No adherence 61.6% (n= 611)	Odds Ratio* (CI 95%) for No adherence (Morisky score >2) No adherence 38.2% (n=379)
**Demographic and socioeconomic characteristic**		
Age	0.99 (0.98 – 1.00)	0.98 (0.97 – 1.00)
Male sex	**1.54 (1.23 – 1.93)‡**	**1.53 (1.22 – 1.92)‡**
Low education (<8 years of education)	1.06 (0.85 – 1.32)	1.19 (0.96 – 1.48)
Low family income (<US$80 per person month)	**1.39 (1.11 – 1.72)‡**	**1.69 (1.35 – 2.09)‡**
**Psychosocial variables**		
Inadequate patient –physician relation	1.04 (0.84 – 1.29)	0.95 (0.76 – 1.18)
High emotional stress-depression score	**2.15 (1.68 – 2.76)‡**	**1.97 (1.56 – 2.49)‡**
Low social support	**1.34 (1.08 – 1.66)‡**	**1.32 (1.06 – 1.63)‡**
Low Knowledge about hypertension	0.98 (0.81 – 1.21)	1.11 (0.91 – 1.36)
**Antihypertensive treatment variables**		
Uncontrolled BP (≥ 140/90 mmHg)	**1.52 (1.22 – 1.90)‡**	**1.85 (1.49 – 2.29)‡**
Multiple antihypertensive RX	0.92 (0.75 – 1.13)	**0.78 (0.63 – 0.96)†**
Time of care in CVHP (years)	0.98 (0.95 – 1.00)	0.98 (0.96 – 1.01)
Systolic blood pressure baseline in CVHP	1.00 (0.99 – 1.00)	0.99 (0.98 – 1.00)
Diastolic blood pressure baseline in CVHP	1.00 (0.99 – 1.00)	1.00 (0.99 – 1.01)
**Anthropometrics and clinical variables**		
Diabetes mellitus	**0.81 (0.66 – 0.99)†**	**0.71 (0.58 – 0.87)‡**
Elevated total cholesterol (≥ 200 mg/dL)	**1.26 (1.04 – 1.54)†**	1.17 (0.96 – 1.43)
BMI (Kg/m^2)	1.00 (0.98 – 1.02)	1.01 (0.99 – 1.03)
**Lifestyle habits**		
Smoking (current smoker)	0.98 (0.78 – 1.22)	1.01 (0.80 – 1.26)
Alcohol related abnormal behavior	1.18 (0.86 – 1.60)	1.28 (0.95 – 1.72)
Sedentary	0.98 (0.75 – 1.29)	**1.40 (1.05 – 1.86)†**

†p <0.05, ‡p <0.01. *OR adjusted for all the variables that were significant at the p<0.05 level in unadjusted model: sex (male), low family income <US$80 per person/month), inadequate patient-physician relation (score ≤ 71), high emotional stress-depression (score ≥7), low social support (score <57), uncontrolled BP (≥ 140/90 mmHg), multiple antihypertensive RX (≥ 2 drug antihypertensive), diabetes, elevate total cholesterol (≥ 200 mg/dL), alcohol related abnormal (score ≥2) as dichotomous variables.
